# Transgender and gender diverse curriculum in medical imaging programs: a case study

**DOI:** 10.1186/s12909-024-05426-x

**Published:** 2024-04-25

**Authors:** Sidsel Pedersen, Lynn Corcoran

**Affiliations:** Athabasca University, Athabasca, Canada

**Keywords:** Medical imaging education, Transgender, Gender diverse, Curriculum, Content development

## Abstract

**Background:**

Transgender and gender diverse (TGD) individuals face barriers, including harassment and discrimination, when accessing healthcare services. Medical imaging procedures require personal information to be shared, such as date of last menstrual cycle and/or pregnancy status; some imaging exams are also invasive or intimate in nature. Terminology is based on binary sex creating an inherently cis-heteronormative environment. TGD patients fear being outed and often feel a need to function as educators and advocates for their care. Incorporation of inclusive healthcare curriculum related to TGD populations is an effective means of educating new health providers and promotes safer and more inclusive spaces in healthcare settings. Educators face barriers which hinder the creation and implementation of TGD content. The purpose of this study was to examine the impacts educators are faced with when creating and delivering TGD content in their medical imaging curriculum.

**Methods:**

A case study of medical imaging programs at a Canadian post-secondary institute was undertaken. Data was collected via semi-structured interviews with faculty. Relevant institutional documents such as strategic plans, policies/procedures, websites, and competency profiles were accessed. Framework analysis was used to analyze the data.

**Results:**

The study found seven themes that influence the development of TGD curriculum as follows: familiarity and comfort with the curriculum and content change process; collaboration with other healthcare programs; teaching expertise; management of course workload and related. duties; connections to the TGD community; knowledge of required TGD content and existing gaps in curriculum; and access to supports.

**Conclusions:**

Understanding educators’ perspectives can lead to an increased sense of empowerment for them to create and incorporate TGD curriculum in the future. Many post- secondary institutions are incorporating an inclusive lens to educational plans; this research can be used in future curriculum design projects. The goal is improved medical imaging experiences for the TGD population.

There is an urgent need to improve medical imaging experiences of transgender and gender diverse (TGD) people. Across healthcare programs, including medical imaging programs, TGD curriculum is inadequate [1–5]. By including TGD curricula in educational programs, graduates will be better prepared to provide safer and inclusive environments for this marginalized group which can potentially lead to better health outcomes. A robust understanding of the barriers that faculty face when trying to develop TGD content is needed to ensure that TGD curricula is incorporated into Medical Radiologic Technology (MRT) education.

## Background

Transgender and gender diverse patients are often faced with discrimination, stigma, microaggressions, and physical violence when seeking medical care [6]. Transgender and gender diverse patients include those patients with gender identities or expressions that differ from the gender socially attributed to the sex assigned to them at birth [7]. Almost 50% of TGD participants in the Trans PULSE Canada [8] survey described having an unmet healthcare need in the past year. Transphobia has been acknowledged as a major barrier for TGD patients in healthcare [9, 10]. To ensure equitable, fair, and safe treatment, healthcare providers must demonstrate entry-to-practice competencies when working with TGD populations [11, 12]. Floyd et al. [13] identified a lack of cultural competence, social stigma, and cis-heteronormative environments as barriers that TGD patients are faced with in radiology departments. A cis-heteronormative environment is based on several assumptions: everyone identifies as the gender they were assigned at birth; those who do not are considered abnormal; and everyone is heterosexual [14]. As such, these environments are unwelcoming for TGD people. A study of transgender and gender nonbinary (TGNB) patient experiences by Grimstad et al. [15] found that 70.8% of respondents reported having at least one negative imaging encounter. In the same study, nearly one-third of respondents had to take it upon themselves to educate staff to receive appropriate care.

A review of TGD curriculum implementation in medical imaging programs found that educators acknowledged the importance of adding sexual and gender minority content to curriculum, however they lacked training, resources, and institutional support to develop appropriate content [16]. There is a gap in TGD content and curriculum in medical imaging programs, but there is also a lack of focus on why educators are not incorporating this type of content into their courses [16]. This study will lead to a deeper understanding of this issue. Ultimately, this study contributes to the understanding of development and delivery of TGD education in medical imaging programs as well as having the potential to improve the patient experience for TGD people seeking care in the health system.

## Methods

Case study methodology was used in this study to facilitate development of theory, evaluation of programs, and development of interventions [17]. This method is also fitting for early, exploratory research where there is minimal understanding of the phenomena [18]. The aim of this study was to examine the impacts educators are faced with when creating and delivering TGD content in their medical imaging curriculum. The setting for this exploratory single case study was a polytechnic post-secondary institution in western Canada. A polytechnic institution uses an experiential learning model whereby learners engage in theoretical learning combined with applied, practical skills. The diagnostic imaging portfolio consisted of three medical imaging programs: Diagnostic Medical Sonography (DMS), Medical Radiologic Technology (MRT), and Nuclear Medicine Technology (NMT).

### Data collection

Data was collected from two sources: semi-structured interviews and institutional documents.

#### Interviews

Semi-structured interviews were conducted in-person and online with nine faculty members from the following imaging programs: DMS, MRT, and NMT. The study was introduced to potential participants during a medical imaging faculty meeting. A follow-up email was sent to faculty with a recruitment poster containing information including details related to the study such as anonymity, confidentiality, and safe keeping of data. Semi-structured interviews were conducted using an interview guide; they ranged from 30 to 50 min in length. Interviews were digitally recorded and transcribed using Otter.ai. Participants were given the opportunity to review their transcripts. Following participants’ approval of their transcripts, identifiers were removed to ensure anonymity. This study was approved by the Athabasca University Research Ethics Board and the Research Ethics Board of the polytechnic post-secondary institution in which the research took place.

#### Institutional and disciplinary documents

Current post-secondary institutional documents as well as documents relating to the MRT discipline were collected. This data was collected because in case study methodology, use of multiple data sources enhances the reliability of the study [19]. Institutional documents providing details regarding the equity, diversity, and inclusion (EDI) strategy of the post-secondary polytechnic institution were accessed and reviewed. Additionally, a review of the polytechnic’s social media platforms was undertaken. Program specific documents linked to curriculum development were also accessed and reviewed. Disciplinary documents from the national professional association and national accrediting body related to MRT such as competency profiles were another data source. See Table 1 for a list of institutional and disciplinary documents.

**Table 1:** Document Review

Institution Documents	
EDI Strategic plan	Equity, Diversity and inclusion (EDI) strategic plan
Strategic plan	New World. New Thinking 2020–2025
Annual report 20/21	2020/21 Annual Report
**Institution Policies & Procedures**	
curriculum review	AC 2.24.1
course outline and maps	AC.2.24.2
**HPS website**	https://www.sait.ca/sait-schools/school-of-health-and- public-safety
**Institution EDI website**	
landing page	https://www.sait.ca/about-sait/equity-diversity-and- inclusion
Equity, Diversity and Inclusion Training	https://www.sait.ca/about-sait/equity-diversity-and- inclusion/edi-training
EDI Strategy 1st year anniversary	https://youtu.be/sZciPnsjtPU
Inclusion talks	https://www.sait.ca/about-sait/equity-diversity-and- inclusion/inclusion-talks
Understanding pronouns	https://www.sait.ca/about-sait/equity-diversity-and- inclusion/understanding-pronouns
PERS 148: Introduction to Effective Intercultural Communication	https://forms.office.com/Pages/ResponsePage.aspx?id=gy Ev9Wef0kq2Vm91T- GWy6VqxMQzm31MmY3nlDDzqihUN09ENkI1MjYwN0k4RV lQVVJOSzc4SkQ3Mi4u
**Pride**	
landing page	https://www.sait.ca/student-life/pride-at-sait
How to be an Ally	https://youtu.be/xS5FMErj0SE
Universal washroom	https://www.sait.ca/assets/images/sait/in-body-and- galleries/student-life/in-pride-campus-map-45 × 582.jpg
**Competency profile**	
NEW CAMRT MRT’s	https://www.camrt.ca/wp- content/uploads/2021/10/National-Competency-Profile- 2019.pdf
Current CAMRT MRT (NM)	https://www.camrt.ca/wp- content/uploads/2018/08/Modified-NM-Profile-Final-.pdf
Current CAMRT MRT (R)	https://www.camrt.ca/wp- content/uploads/2018/08/Modified-Rad-Tech-Profile- Final.pdf
Sonography Canada	https://sonographycanada.ca/app/uploads/2021/10/Sonog raphy-Canada-NCP-6.1-EN-for-website-10.20.2021.pdf

### Data analysis

Framework Analysis was used in the analysis of this case study. It is a practical approach that provides a way to manage multiple data sets, including interview transcripts and documents [20]. The overall objective of Framework Analysis is to identify, describe, compare, and contrast key patterns and themes [20]. The five stages of Framework Analysis are data familiarization, framework identification, indexing, charting, and mapping/interpretation.^20^ During data familiarization, immersion in the raw data was completed by reading interview transcripts and documents. Key themes and samples illustrating these themes were tentatively identified. Coding was done in the framework identification stage [20]. Interview transcripts, institutional and disciplinary documents were coded with the goal of building a functioning framework whereby the themes were conceptually related to one another. Indexing involves an iterative process of systematically linking the framework to the data [20]. During this stage, categories and subcategories were developed across the data sets and a systematic indexing of this data was established. The charting process involves a considerable amount of abstraction and synthesis while facilitating a summary of the indexed data [20]. With the formation of charts, indexed themes were categorized. During the final stages of Framework Analysis, mapping and interpreting, comparison of potential areas of interest was completed by examining variations within the categories and subcategories while looking for clusters of data [20]. Interpretations demonstrated associations and relationships across the data sets.

## Results

Two major categories were identified: Individual Attributes and Collective Influences. Individual attributes are qualities held by the educator that impact their ability to develop and delivery TGD content including career choice and educational background; knowledge of and comfort with the curriculum and content change process; awareness of and personal experiences with the TGD community; and an inherent inquiry mindset. Collective influences address the impact that the post-secondary institution has on the individual attributes including visibility of EDI strategies; onboarding processes; and collaboration with healthcare system partners.

### Individual attributes

#### Career choice and educational background

The reasons participants chose a career in medical imaging and their educational background revealed similarities and differences. Participants indicated biology and anatomy were interesting and that they had always felt they wanted a career in healthcare but not as a physician or nurse. Medical imaging was suitable for those that desired something with a technical focus, yet still allowed for direct patient contact. Though the initial rationale for choosing medical imaging as a career may have had some similarities, the pathway was often unique. For seven of the nine participants interviewed graduating from a medical imaging program was not their first post-secondary experience.

The transition from working clinically as a medical imaging technologist to becoming an educator was also worthy of exploration. Participants mentioned that they had enjoyed working with students in the clinical setting as a technologist, and often gravitated towards formal preceptor roles. They reflected on their own time as a student and remembered instructors that were positive role models. Educators entered the classroom setting with various personal and professional backgrounds and these individual attributes were reflected in their teaching.

#### Knowledge of and comfort with the curriculum and content change process

Knowing how instructors were informed of curriculum changes and how these changes were implemented in the classroom, labs, or in practicum was important in understanding how instructors thought about and carried out these changes. Differentiating curriculum and content was a starting point. Participants described curriculum as the official learning objectives and outcomes of the course. The curriculum is housed in course outlines which were identified as legal contracts between the students and the post-secondary institution. Accreditation standards and entry-to-practice competencies were referred to when participants explained the curriculum in their own words. Curriculum was described as rigid and onerous with the recognition that it was slow to change and there were processes and timelines for doing these revisions. Alternately, participants described content more dynamically in terms of their teaching practice; content was easy to change and flexible. Legacy content was defined as content from previous course deliveries that was passed down from one instructor to the next. Some participants relied heavily on legacy content for the first delivery of a course offering while others felt more comfortable creating their own content.

With institutional documents, there were policies and procedures pertaining to curriculum review and course outlines, which provided guidance to instructors regarding the content change process. Some instructors mentioned that they gained experience with these processes as part of program redesigns; not all participants had been part of that process. Participants mentioned that curriculum changes were supported, when necessary, but they often felt making content changes could be done with little oversight and as required for course improvement. Overall, the participants understood the importance of curriculum and why the lengthier process to change was required.

#### Awareness of and personal experiences with the tgd community

Many participants had personal experiences with TGD populations and reported knowing general information about the community. Participants mentioned that concepts such as use of pronouns, gender neutral washrooms, and inclusive space signage were more visible in public spaces such as bookstores, coffee shops, and restaurants. Participants that had personal relationships with a TGD community member were empathic to the negative experiences of these people in the healthcare system.

Participants also sensed a change in the student body on campus. More students openly identify as being part of the 2SLGBTQ + community and participants noted they had students in their classrooms that belonged to the community. The participants reflected on ways that they had begun making their classrooms safer and more inclusive spaces. One participant mentioned asking students to provide names and pronouns during introductions to the lines of communication to any student that may need it.

#### Inherent inquiry mindset

An inherent inquiry mindset involves the desire for continuous learning and professional growth; this was identified as an attribute of participants in this study. This mindset was one reason for their switch from being a practicing technologist in the healthcare system to becoming a faculty member at an educational institution. This was evident by statements such as:

*“I always have desire to learn new things;” “I need to learn the content more in depth before teaching it in the classroom;” “I am a lifelong learner;” “I want to know more about learning theories;”* and *“when there is a lack of current resources I do further digging to learn more.”* There was a clear theme that the desire and need for continuous learning was seen as a benefit to the role of being an educator. Many of the participants expressed the need for continuing their professional growth as well-suited to working in an educational institution with structure and processes.

The visibility of the inherent inquiry mindset theme was also supported in several of the institutional documents. Within the strategic plan there was mention of commitment to excellence where recognition and support of employee excellence is highlighted by way of “hire for a growth mindset curiosity and collaboration.” [21, p. 4]. A recent job posting within in the DI portfolio also alluded to an inherent inquiry mindset as an attribute for the position: “Demonstrates a positive and respectful attitude and has a growth mindset; Committed to quality and engages in continuous improvement; Reflective about personal and professional growth.” [22, p. 4].

### Collective influences

#### Visibility of EDI strategies

A key factor for participants was the strength and visibility of an institutional EDI strategy. Participants indicated that there was a sense that the institution prioritized EDI initiatives across campus, and in the document review and interviews it was evident that all institutional levels were engaged with the EDI strategic plan.

#### Onboarding processes

Many participants reflected on their transition from working in the healthcare system to becoming a faculty member. There were several indications of formalized institutional onboarding including structured courses and mandatory faculty training. Participants had unique experiences with the onboarding process and for some there was minimal time between hire date and start date in the classroom, which led to minimal onboarding being completed.

#### Collaboration with healthcare system partners

It was evident that close collaboration with healthcare system partners is required for the programs to achieve and maintain accreditation standards. The relationship between partners and faculty is maintained through a variety of committees. There is a focus on a continuous improvement process to ensure that program curricula meet the needs of healthcare system partners as well as the national competency profiles.

### Revision of the research question

As is common in case study research methodology, ongoing examination and interpretation of the data led to refinement of the foundational research question. The revision of the research question is required to ensure that it remains focused and within the scope of the study [15]. In this study the research question was revised to: *How do individual attributes and collective influences impact educators when they are creating and delivering TGD content into medical imaging programs?*

### Themes

Seven themes were identified: familiarity and comfort with curriculum and content change process; collaboration with other healthcare programs; teaching expertise; management of course workload and related duties; connections to the TGD community; knowledge of required TGD content and existing gaps in curriculum; and access to supports. The seven themes that were identified through the data analysis relate to the influences that impact educators when they are creating and delivering TGD content. A connection was drawn between the individual attributes and collective influences that were identified in the results section. See Table 2.

**Table 2:** Individual Attributes and Collective Influences Related Themes

Themes	Individual Attributes	Collective Influences
**Familiarity and comfort with curriculum and content change process**	• Knowledge and comfort of curriculum and content change process	• Onboarding
**Collaboration with other healthcare programs**	• Inquire mindset	• Visibility of EDI strategies • Onboarding
**Teaching expertise**	• Career choice and educational background • Knowledge and comfort of curriculum and content change process	• Onboarding
**Management of course workload and related duties**	• Knowledge and comfort of curriculum and content change process • Inquire mindset	• Onboarding
**Connections to the transgender and gender diverse community**	• Awareness of, and personal experiences with the TGD community • Inquire mindset	• Visibility of EDI strategies • Collaboration with healthcare system partners
**Knowledge of required transgender and gender diverse content and existing gaps in curriculum**	• Career choice and educational background • Knowledge and comfort of curriculum and content change process • Awareness of, and personal experiences with the TGD community • Inquire mindset	• Visibility of EDI strategies • Collaboration with healthcare system partners
**Access to supports**	• Inquire mindset	• Visibility of EDI strategies • Onboarding

#### Familiarity and comfort with the curriculum and content change processes

Individually, participants mentioned varying levels of knowledge and comfort with the curriculum change process at the institution. Many described that they aligned the content directly with the formal course outline. Those that had taken part in a curriculum review and redesign had a better understanding of how to complete changes. From the institutional perspective, documents revealed that there was a formal policy and procedure in place whereby an annual review of curriculum is expected to be completed by leadership and faculty. The process for changing curriculum can be onerous and follows a rigorous cycle with a strict timeline. The instructors vocalized that more guidance around this process could be helpful when discussing the ability to add TGD content. *“At times, when I would ask for guidance, or help with certain things [changing content], I felt like I maybe didn’t get as much support as I would have liked.”* (Participant #2).

#### Collaboration with other healthcare programs

Participants had little knowledge of what initiatives in TGD content were being implemented outside of their own course, program, or portfolio. There was a reference to subject matter experts from within the institution, but they were not specifically involved in course content development. Participants alluded to the possibility of having the institution support the creation of a core course within the healthcare programs as many patient-centered competencies for the TGD community were transferable among healthcare providers. Having a core course would ensure experts could collaborate to help create and facilitate common competencies and ensure the terminology and messaging was consistent across the programs. *“I wonder if there is a way to go to an expert, and then continue the conversation in our courses”* (Participant #8).

#### Teaching expertise

Instructors at the institution are hired as subject matter experts of their profession and applicants do not require formal teaching education or classroom experience to be hired. A variety of in-house facilitated teaching development courses are offered, to help prepare new instructors for classroom delivery. Courses focus on teaching strategies and delivery of content, *“The course through [institution], which deals with lecture creation, content creation as well as grading and feedback”* (Participant # 4). Some participants had challenges attending some of these offerings due to scheduling conflicts. There were also a few participants who started teaching a short time after being hired and were only able to complete the courses after already teaching in the classroom.

When instructors are assigned new courses to teach, they rely heavily on legacy content. Much of their time is spent learning the material in a way that makes them feel comfortable teaching it in the classroom. The lack of TGD legacy content made it challenging for faculty members to include TGD outcomes.

#### Management of course workload and related duties

Participants often mentioned that the biggest barrier to developing and creating new content of any kind was lack of time, *“We are developing on the fly as we are teaching”* (Participant #3). It was evident faculty felt they were overcommitted and that completing major course updates while managing other work-related duties was a challenge. When instructors were given new courses to teach, they often mentioned that they spent a large amount of time learning the material prior to the start of the course. Participants mentioned that they understood the importance of adding TGD content into the program, but with additional demands of assignable duties and maintaining a work/life balance many did not feel they had the time required to adequately create new content. In the context of limited time, it is important to consider reasons why TGD curriculum is not prioritized by faculty leaders.

#### Connections to the TGD community

All participants were able to accurately define transgender and gender diversity in their own words but often felt unsure that they were using the correct terminology and questioned their own knowledge around the terms. Personal connections to the TGD community varied among the participants; some mentioned having family members, relatives, or friends who identify as part of the TGD community, others felt there was an increase in their awareness based on social media influences and mainstream media. Many participants were open in sharing some of the experiences that they have encountered and how those experiences influenced their current practice inside and outside of the classroom. “I feel free to add it [inclusive teaching practices] *because it matters to me, but there has not been a very strong push* [from leadership] *that you have to add it”* (Participant #4).

There was an expressed interest to learn more about the TGD community and how, as faculty members in a cis-heteronormative environment, they could better support and reflect the needs of the TGD community authentically in their courses. During the interviews, participants recognized that an authentic connection to the community was a key factor in learning about the community and their needs in the healthcare setting. “*You can do a lot of work as an ally, but there is nothing like the experience of a person who is living it*” (Participant #4).

#### Knowledge of required tgd content and gaps in existing curriculum

During the interviews, there were challenges in defining what was meant by the term “TGD content.” It was clear that there was an overwhelming sense that the programs did not currently include specific TGD content. Participants identified courses in which they believed TGD content could be included. Professional practice was a suitable course in which topics related to TGD patients could be added.

All programs work very closely with healthcare stakeholders to ensure that students are well prepared to enter the workforce. Some participants mentioned that they were interested in incorporating TGD content into their courses despite the lack of prompting from healthcare system partners. This deviated somewhat from the standard that is often seen in programs where incorporating changes to content mimics changes in healthcare (as seen with advancements in technology or updates of protocols). There were concerns expressed that if students were taught new standards in the program that they might experience some pushback from healthcare partners when entering practicum.

#### Access to supports

Several participants expressed a desire for access to additional resources and supports with TGD content in the classroom: *“You can read, and you can read, and you can read. It would be nice with a facilitated workshop”* (Participant #7). Many participants mentioned that they had some access to resources required to increase their own knowledge about general EDI topics, but limited access to specific resources dedicated to the TGD community. These supports were often through professional bodies and open-education resources with other post-secondary institutions.

Instructors did not feel that they had enough knowledge and expertise to teach this TGD content in the classroom. There was fear of saying something wrong and unintentionally offending people. The institutional EDI strategic plan included assurances the resources would be available to update curriculum. During the interviews many participants were unaware of any support currently in place within the institution that could help them in their desire to create more TGD content and inclusive classrooms. *“That is why we are in the healthcare field is to make patients feel better in a really difficult time in their lives and you can’t really accomplish that task if you are ignorant”* (Participant #10).

## Discussion

Transgender and gender diverse content in healthcare curriculum is a novel topic of study. Studies related to increasing EDI content in nursing, medicine, dentistry, pharmacy, and social work can provide further insight for institutional curriculum reforms relating to TGD content [23–27]. In this study, individual attributes and collective influences converged to influence TGD content and curriculum development in medical imaging programs. Themes emerged linked to specific roles associated with both institutional supports and the individual faculty members; this finding is supported by previous research [23, 28].

### The role of the institution

The significance of institutional support should not be down-played, as without.

leadership support many initiatives and diversity projects would not be possible [23, 27]. Perceived ambivalence from leadership or a lack of institutional oversight to include diversity topics impedes faculty members’ abilities to effect change [24]. Ensuring that all faculty members are provided with resources and training to increase their confidence in these topics is imperative; faculty members report feeling unprepared and worried about using incorrect terminology with students [24, 27]. It is important to prioritize accessibility of resources by providing support for faculty to attend education sessions. In this study, participants mentioned that the visible EDI strategies on campus, and support from leadership around various EDI topics encouraged them to work toward development of content and curriculum. A robust institutional strategy can ensure that TGD content is integrated across all program courses as part of the instructional design. Use of consistent language throughout course outlines is imperative. Instructors rely heavily on the course outlines when updating their course content. When updated and consistent language and terminology is used in course outlines, it is more likely this will be replicated in the course content.

### The role of faculty

Faculty members have a perceived lack of knowledge and expertise on EDI topics which is a barrier to development and incorporation of EDI content [23]. If there is only a single faculty member that has the skills and knowledge related to EDI, they are left isolated and carry a large burden of responsibility similar to Indigenous faculty members solely teaching Indigenous content [24]. Mentoring programs and multi-professional education opportunities can increase awareness and strengthen relationships between faculty members and in turn build competence and confidence around EDI content development [29, 30]. Engaging with TGD community members through conversations, mentoring programs, or simulation scenarios can lead to faculty members gaining an improved sense of connectedness to the community and can expand their knowledge and comfort level [29, 31, 32]. 

The themes in this study are not positive or negative. This is a complex and layered issue and as such, the themes should not be considered an itemized list that can be addressed. The process of addressing barriers to inclusion of TGD content in the curriculum is iterative. With deliberate strategies in place, the capacity of faculty members is developed related to TGD content and care of TGD patients. Over time, the curriculum reflects this development in knowledge. Ultimately, this shift is actualized in the students’ clinical practice with the goal of improving care of TGD patients.

### Limitations

There are several limitations related to this study. The definition of a “case” and the approach to undertaking case study research might be considered limitations because there is contention among scholars as to what constitutes a case as well as to the various theoretical traditions used to examine a particular case [33]. The case for this study involved a medical imaging faculty in a single post-secondary polytechnic institution in Canada which represents a limited sample both in size and diversity related to the identities of participants being interviewed which limits generalizability. The inclusion of several post-secondary institutions across health disciplines would be a compelling next step for future research in this area. In addition, this study was limited to TGD curriculum; it did not include an exploration of development of medical imaging curriculum for intersex people. This topic is also worthy of further study.

### Implications

This study highlights the medical imaging educator’s perspective of the influences that currently preclude the development of TGD curriculum. This understanding can lead to an increased awareness of facilitators and barriers for educators to consider when developing TGD content in their courses. Many post-secondary institutions are incorporating an inclusive lens to their strategic plans; this research can be used as a resource for future curriculum design projects. Ensuring that the curriculum is updated will not only help meet accreditation requirements with current national competency profiles but may promote an inclusive learning environment for students. Including this curriculum in medical imaging programs will better prepare graduates in creating an inclusive and safer space for TGD patients in medical imaging settings. From an educational perspective, this study can serve as a guide for increasing awareness of and overcoming factors that currently impact faculty members in creating and delivering TGD. Further commitment and support from leadership will ensure that faculty members feel supported with including and developing TGD content in their courses.

This study also highlights the need for further research in this area. Many of the.

influences that impacted faculty in this study can be linked to the post-secondary polytechnic institution in which it was conducted. This includes the onboarding processes, policies, and procedures as well as institutional strategic plans. To gain a better understanding of these and other barriers similar case studies could be completed at other post-secondary institutions. Completion of a multi-site case study could also be warranted to compare results from medical imaging programs nationally.

## Conclusion

This case study was undertaken with the goal of learning more about what impacts an educator is faced with when creating and delivering TGD content in their courses. Collective institutional support is a key factor for enabling individual educators to create and deliver TGD content. These supports begin during the onboarding phase of new faculty, and should include information about curriculum development process, EDI strategies, and additional learning opportunities where faculty members can increase their knowledge and connections with the TGD community. Though appropriate and sustained leadership support can be seen as a catalyst for change, the execution and work must come from the entire faculty. A comprehensive approach where all levels of the institution work together is required to overcome the barriers that currently preclude incorporation of TGD curriculum.

## Data Availability

The dataset related to documents from the public domain retrieved and analyzed during the current study are available from the corresponding author on reasonable request. The dataset related to interview transcripts generated and analyzed during the current study are not publicly available due to ethical approval. Contact Sidsel Pedersen/sidsel.pedersen@sait.ca to request the data from this study.

## Declarations

The protocol and consent form were reviewed by the SAIT Research Ethics Board and were found to be acceptable on ethical grounds for research involving human subjects. Informed consent to participate in the study was obtained from al participants.

### Authors’ information

Authors and Affiliations.

Sidsel Pedersen, School of Health and Public Policy, Southern Alberta Institute of Technology Polytechnic, Canada.

Lynn Corcoran, Faculty of Health Disciplines, Athabasca University, Canada.
